# A Computational Geometry Generation Method for Creating 3D Printed Composites and Porous Structures

**DOI:** 10.3390/ma14102507

**Published:** 2021-05-12

**Authors:** Petros Siegkas

**Affiliations:** Department of Engineering, Nottingham Trent University, Clifton, Nottingham NG11 8NS, UK; petros.siegkas@ntu.ac.uk

**Keywords:** additive manufacturing, 3D printed material characterization, two phase composites, porous materials, Voronoi cells

## Abstract

A computational method for generating porous materials and composite structures was developed and implemented. The method is based on using 3D Voronoi cells to partition a defined space into segments. The topology of the segments can be controlled by controlling the Voronoi cell set. The geometries can be realized by additive manufacturing methods, and materials can be assigned to each segment. The geometries are generated and processed virtually. The macroscopic mechanical properties of the resulting structures can be tuned by controlling microstructural features. The method is implemented in generating porous and composite structures using polymer filaments i.e., polylactic acid (PLA), thermoplastic polyurethane (TPU) and nylon. The geometries are realized using commercially available double nozzle fusion deposition modelling (FDM) equipment. The compressive properties of the generated porous and composite configurations are tested quasi statically. The structures are either porous of a single material or composites of two materials that are geometrically intertwined. The method is used to produce and explore promising material combinations that could otherwise be difficult to mix. It is potentially applicable with a variety of additive manufacturing methods, size scales, and materials for a range of potential applications.

## 1. Introduction

Additive manufacturing is becoming more accessible and with an increasing variety of materials. Additive can be a cost efficient alternative to subtractive manufacturing as it can reduce the amount of waste material and produce customized and complex designs [1]. Similarly porous materials and structures could be produced by means of additive manufacturing. However the limitations in terms of resulting material behavior could be restrictive in the use of 3D printing for functional parts with specific mechanical property requirements.

3D printing has been used to produce porous structures as biological cell scaffolds in tissue regeneration applications [2,3,4,5]. The produced structures can be heterogeneous with functionally gradient porosity [6] and are often regular in terms of pore shape [7,8]. Porous scaffolds tend to be insufficiently similar to bone pores and are mechanically weak [9]. Obtaining exact bone geometry through X-ray micro-tomography can be expensive and is not always available [9,10]. The geometrical and mechanical properties can be relevant to cell development e.g., enhanced with applied mechanical stimuli. It has been found that the introduction of small strains during bone growth enhances the tissue development [11,12]. The control of the mechanical properties of implanted cell scaffolds is important both in providing structural support but also in regulating the amount of strain that can be accommodated.

3D printed structures are also considered for controlled drug release applications with the use of impregnated bioresorbable materials [13,14]. Complex 3D printed porous scaffolds could also be suitable for creating biomimetic structures that could replicate or provide a realistic tumor cell microenvironment for 3D in vitro studies of cancer cells [15].

Furthermore, new materials and technologies offer promising opportunities in designing better protection equipment. Strain rate sensitive materials suitable for additive manufacturing techniques [16] can be combined with intelligent designs to enhance impact energy absorption. Lightweight lattice structures can be designed to absorb substantial amounts of energy and outperform conventional materials [17,18,19]. The additive manufacturing techniques enable the design of custom and personalized equipment. Furthermore, different materials can be combined to produce composites that could work synergistically for specific applications. The mechanical properties of 3D printed lightweight cellular composites can be controlled by controlling the design and print process that can affect their elastic properties and strength [20]. This possibility could be useful in applications including prototyping, small scale production, and custom functional parts.

Computer aided design, using stochastic strategies, has been previously used to represent porosity [21]. The Voronoi tessellation has also been used in a variety of applications. Extruded 2D Voronoi cells can be mapped on 3D surfaces to resemble nacre structures in biomimicking composites [22]. Complex antennas based on the Voronoi tessellation can be 3D printed [23], and high porosity Voronoi 2D patterns can be optimized and used as low density fillers [24]. 3D Voronoi cells have been used to virtually represent high porosity and using the edges as struts [25]. Voronoi cells following a size distribution and with a proximity criterion were used with finite element modelling for predicting material properties of porous structures [26,27].

This study features a method using 3D Voronoi cells as space holders for generating medium density porous structures and composites. The properties and geometrical features can be controlled to fit specific applications e.g., to mimic bone microstructure. Combinations of materials that might be difficult to mix using traditional methods, could be combined structurally in intertwined configurations. The method is applied using three materials i.e., PLA, TPU and Nylon, and can potentially be used with various additive manufacturing techniques, materials, and at different scales.

## 2. Materials and Methods

### 2.1. Virtual Geometry Generation

**Figure 1:**
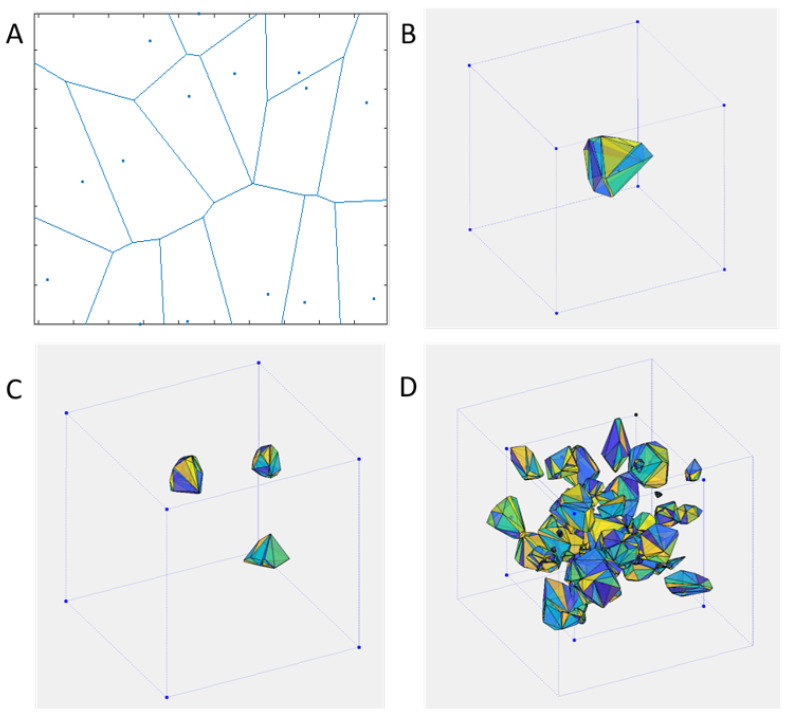
(**A**) 2D Voronoi cells, (**B**) 3D Voronoi cell, (**C**) Voronoi cells as pores produced by randomly generated seed points, (**D**) differently sized Voronoi cells produced iteratively.

The geometries were generated using a method based on the Voronoi tessellation. In two dimensions a Voronoi diagram partitions a plane into regions around seed points (Figure 1A). Each region encloses all points that are closer to its seed point than to any other. In three dimensions the regions can be enclosed by polyhedral cells (Figure 1B). The size and shape of these cells within a defined volume i.e., a cube, depends on the density and position of the seed points (Figure 1C).

For the purposes of this study, a number of randomly generated points were placed within a cube space. The cube was then partitioned to an equal number of polyhedral cells. A percentage of those cells was exported, so as to approximately correspond to the desired porosity for porous materials, or the ratio between materials for composites. A Boolean operation was applied to subtract those cells from the initial cube volume, hence leaving behind gaps that would correspond to either pores or space for a material other than the rest of the volume.

Topologically the space could be defined as below [28]:

A number of bounded Xi volumes (Voronoi cells), form a set called X where $X=(\cup X_{i})$ and $X_{1}{,\;X}_{2}{,\;X}_{3}....{\;X}_{n}\;\subseteq \;R^{3}$

The set X: is bounded by an external surface B that contains a volume $V_{B}$ where $X_{i}\subseteq V_{B}\forall i$.

The space Y is defined by Equation (1) and demonstrated in

(1) $$Y=V_{B}-(\cup X_{i})\;\mathrm{where}\;Y,\;V_{B}\;\subseteq \;R^{3}$$

The average cell size would correspond to

(2) $${\bar{V}}_{p}=\frac{V_{B}}{i}$$

where ${\bar{V}}_{P}$ is the average pore size, $V_{B}$ is the specimen volume size, i is the number of seed points.

**Figure 2:**
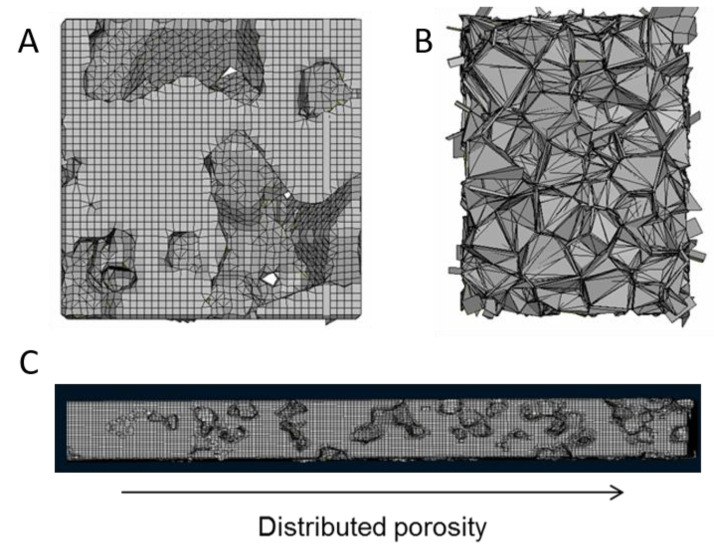
(**A**) porous structure produced by Voronoi cells following a size distribution, (**B**) closed cell foam produced by adding thickness to 3D Voronoi cells, (**C**) porous structure with varying porosity.

The method offers flexibility in terms of the produced geometries. Different cell sizes could be combined in order to approximate a desired cell size distribution (Figure 1D) by repeating the process mentioned above with a different number of seed points, and keeping as many cells as necessary from each repetition so that to accumulate to the desired volume. A radial proximity criterion can applied in combination to Boolean operations to avoid overlapping. Alternatively, geometries with gradual porosity can be produced, or closed cell foam geometry using all the Voronoi cells. The method also offers flexibility in producing a variety of potential structures (Figure 2) [26].

### 2.2. Specimen Preparation

**Figure 3:**
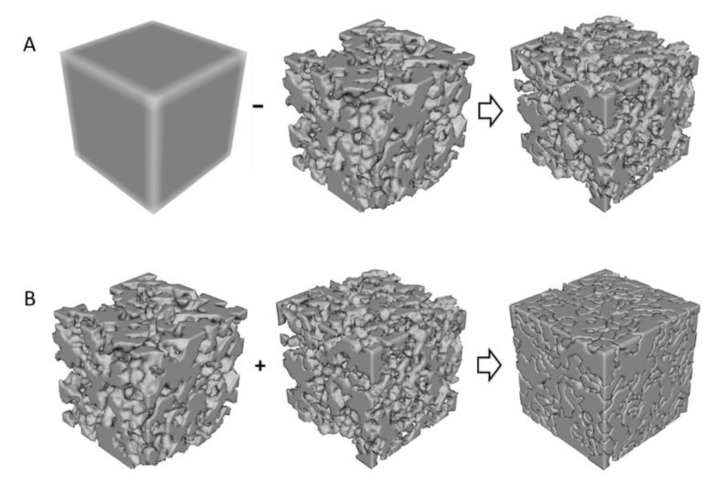
(**A**) Boolean operation producing porous geometry specimens, (**B**) producing composite structure specimens.

The specimens were cubic of approximately 30 mm side. The cubic space was seeded with 5000 points producing an equal number of polyhedral cells. Half of those cells (i.e., 2500) were then subtracted from the volume producing two segments i.e., the set of cells and the rest of the volume at approximately 50% ratio. The process was repeated three times to produce three different geometries with two distinct segments for each (Figure 3). The geometries were imported in 3D printing software, Ultimaker Cura^®^ v.3 (Ultimaker, Cambridge, MA, USA). Ultimaker 3 dual nozzle (0.4 mm) FDM printers were used with three filament materials from the same manufacturer i.e., PLA, TPU, and Nylon. The materials were assigned to the different segments of each geometry and used to produce either porous or composite structures. The manufacturer’s recommended settings were used for each material (Table 1). Support material was not used for the porous structures. Filament retraction during the printing process was disabled for TPU. Each of the three virtual geometries was used to produce four specimens i.e., two porous and two composites resulting in twelve specimens.

### 2.3. Testing

Specimens were tested under quasi static compression at a strain rate of $\dot{\varepsilon }<$10^−3^ s^−1^. A Shimadzu AG-X testing machine was fitted with a 50 KN load cell and used for the testing. The recorded stroke displacement was corrected using the measured machine compliance. Specimens were compressed in the Z axis direction i.e., the axis in which each layer was added during the manufacturing process. Each test was repeated three times using different specimens

## 3. Results-Discussion

**Figure 4:**
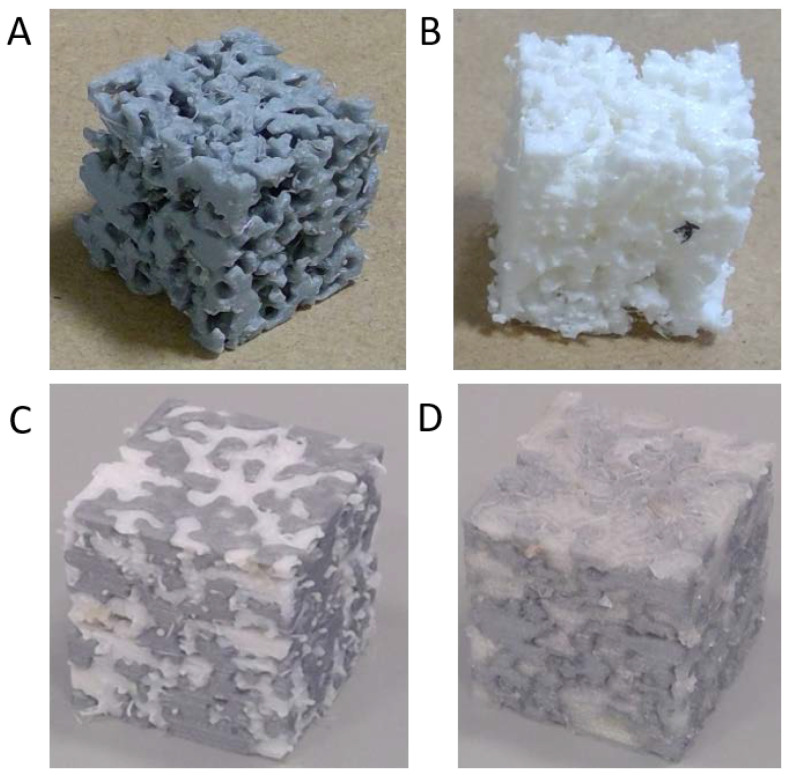
Cube specimens (side of approx. 30 mm) (**A**) 3D printed porous PLA specimen, (**B**) 3D printed porous TPU specimen, (**C**) 3D printed PLA and TPU specimen, (**D**) 3D printed PLA and Nylon specimen.

Three cubic geometries were generated by the method described above. Each geometry was split on two segments of approximately equal volume and materials were assigned for each segment. The produced materials were porous PLA (Figure 4A), porous TPU (Figure 4B), PLA and TPU composite (Figure 4C) and PLA and Nylon composite (Figure 4D). The materials in the composite configuration were intertwined within the structure. An infill setting of 20% was set during the printing process. The resulting specimens were within a density range of approximately ρ = 500–1000 kg·m^−3^.

**Figure 5:**
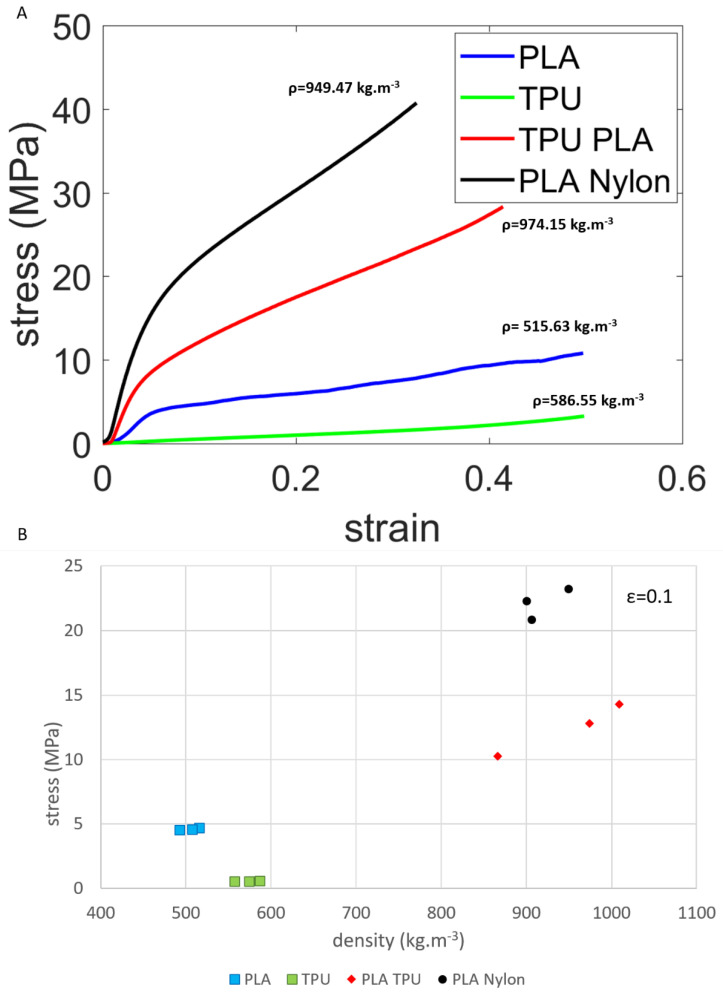
(**A**) Nominal stress and strain compressive response of porous PLA, porous TPU, PLA and TPU composite, PLA and Nylon composite. (**B**) Stress at 0.1 strain and density for the tested specimens.

Figure 5A shows the compressive stress strain behavior profile of the tested specimens. Composite and porous PLA specimens exhibited an elastic plastic behavior i.e., a steep elastic part followed by plastic region of lower gradient. TPU specimens were of significantly lower stiffness.

Figure 5B shows the stress response at 0.1 nominal strain in relation to density for each of the tested specimens. The same three geometries were used for all specimens but with different materials. Density and stress response appears to be more consistent in the porous specimens rather than the composite.

Table 2 provides with the values of the stress response at 0.1 and 0.2 nominal strain in relation to the resulting density of the specimens. The relation of density to stress response in the porous structures and by comparison to fully solid specimens found in literature, appears to be non-linear [29,30].

The combination of soft and hard material in multiphase materials (e.g., TPU and PLA) can result in synergistic effects. Similar approaches in literature, using regular structures, found that while the hard phase can endure larger portion of the load, the softer phase can confine cracks. This could be promising in applications that require damage tolerance and vibration damping. Additionally, phase topology can have an important effect on mechanical properties [31]. Material combinations can also add to the efficiency of the materials. The stress response of the TPU-PLA composite can be more than twice the sum of the stress response that the two porous materials have when used separately (Table 2), and for less than the sum of their densities.

The same three geometries were used for all specimen types. The consistency in the density and stress response of the produced specimens varied between types (Figure 5B). The produced porous structures were generally more consistent in the resulting density, rather than the composite specimens of which PLA—Nylon seemed to be more consistent than PLA—TPU. This might have been due to equipment limitations, such as resolution, nozzle size, in combination with geometrical features of the structures and the specific materials. Infill percentage was set to 20%, however this might not have been consistent between all cross-sections and materials. Cross-sectional variation has been found to affect the resulting properties and possibly infill, which should be considered if scaling is applied [32].

## 4. Conclusions

The study describes and implements a computational method for generating porous materials and composites for additive manufacturing techniques. The method allows for potentially using available materials to create hybrid metamaterials with tailored properties. For the purposes of this study, three geometries were generated and each was split into two segments. Materials were applied either in one segment for porous structures, or in both segments for composites. PLA, TPU, and Nylon were used to generate four types of specimens i.e., porous PLA, porous TPU, PLA and TPU composite, and PLA and Nylon composite. Twelve specimens were produced in total, and tested under quasi static compression.

Two types of porosity were included in the porous materials i.e., porosity introduced by the Voronoi cells and partial infill within the solid parts. Similarly, the composite structures also included partial infill for both materials. Material combinations that could otherwise be difficult to achieve using traditional methods were geometrically intertwined to produce different properties. The use of Voronoi cells results in complex geometries with a potentially large interface surface area that allows for the materials in composites to intertwine in a stable manner. Additionally, stochastic porous structures that can resemble organic configurations may be suitable for cell scaffold applications [9].

The method provides a number of variables that can be potentially manipulated to define the macroscopic material properties. The number, size and positional distribution of the Voronoi cells, the infill percentage during 3D printing, and the combination of materials could provide a range of resulting properties. Material combinations can also have synergistic effects e.g., in damage and vibration management [31].

Further study is required to fully characterize the resulting materials, and determine the range in achievable properties and limitations of the method. Additionally the effect of surface roughness and interface friction in composites could be studied. The number of material combinations is limited by the capability of the additive manufacturing equipment, however the method could also potentially be used with other types of materials (e.g., metals, ceramics, bio printing gels), additive manufacturing methods and size scales. This could potentially find applications including heat exchange, permeability, acoustics, or be used with conductive materials as part of smart material systems. These would be the topics of future work.

## Figures and Tables

**Table 1:** Printing settings.

Settings	PLA	Nylon	TPU
Printing temperature (°C)	200	245	223
Layer height (mm)	0.1	0.1	0.1
Printing speed (mm·s^−1^)	70	70	25
Filament (mm)	2.85	2.85	2.85
Nozzle (mm)	0.4	0.4	0.4
Infill (%)	20	20	20

**Table 2:** Specimen densities and nominal stress response at 0.1 and 0.2 nominal strain.

Material	Density (kg·m^−3^)	Stress (MPa) at 0.1 Strain	Stress (MPa) at 0.2 Strain
PLA-Nylon composite	949.47	23.25	31.39
	905.79	20.87	29.74
	900.27	22.32	32.54
PLA-TPU composite	974.15	12.79	18.03
	866.64	10.27	15.21
	1.01 × 10^3^	14.28	20.55
Porous TPU	586.55	0.59	1.031
	574.09	0.54	0.97
	557.39	0.55	0.94
Porous PLA	515.52	4.7	6
	507.44	4.6	6.7
	492.80	4.55	5.98

## Data Availability

The raw/processed data required to reproduce these findings can be shared upon request and within reasonable time. These cannot be shared at this time due to time limitations.
